# Technology-Facilitated Sexual Harassment Against Women and Psychological Dysfunction: A Test of Objectification Theory

**DOI:** 10.1177/10778012231177998

**Published:** 2023-06-04

**Authors:** Casey Oliver, Shayna Cummings, Erika Puiras, Dwight Mazmanian

**Affiliations:** Department of Psychology, 7890 Lakehead University, Thunder Bay, Canada

**Keywords:** sexual harassment, technology, women, self-objectification, psychological functioning

## Abstract

Objectification theory was tested to examine the potential mediating role of self-objectification in the relationship between technology-facilitated sexual harassment (TFSH) and psychological functioning in a sample of women (*N* = 481). The results indicated that TFSH was associated with eating pathology, alcohol use, and sexual functioning. Furthermore, partial support for the objectification theory was achieved, with self-objectification potentially explaining the relationships between TFSH and eating pathology, and TFSH and alcohol use. This research may shed light on the role of objectification processes in the context of TFSH in women, as well as inform mental health interventions for women victims of TFSH.

Sexual harassment that is perpetrated through technology, also known as technology-facilitated sexual harassment (TFSH), is a form of sexual violence that has emerged in the past decades due to the advent of technology. It can be defined as unwanted sexual attention that is received through technological mediums that, as outlined by Henry and Powell (2018), fit with other sexually violent behaviors under the umbrella term technology-facilitated sexual violence (TFSV). TFSH appears to be one of the more common forms of TFSV, as well as one of the most researched (Henry & Powell, 2018). Although much research on TFSV has targeted TFSH, there still is an overall lack of knowledge about this form of TFSV.

Similar to sexual harassment that occurs in person, early research in the area has indicated that TFSH disproportionately affects women (Douglass et al., 2018). In a study by Cripps and Stermac (2018), TFSH was reported by 53% of their sample of young Canadian women, indicating that one in every two undergraduate student women may experience TFSH in Canada. Similarly, a study by Cuenca-Piqueras and colleagues (2020) demonstrated that TFSH may be experienced more by young women (i.e., those under 30 years of age) than older women, which is in line with the results from research on sexual violence that occurs in-person (Rotenberg, 2017).

The online disinhibition effect is useful for understanding why women may be more vulnerable to TFSH than men (Barak, 2005; Suler, 2004). This effect explains how women are often targeted on technological platforms because of the reduced power that they hold in patriarchal societies (Penny, 2013). This power imbalance between women and men provides an opportunity for those who desire power and control to take advantage of the technological space (e.g., anonymity, lack of legal ramifications) to exert violence (Barak, 2005). This all demonstrates why TFSH should be examined with a gendered lens within a spectrum of acts of violence against women.

Despite the rich body of research that exists on sexual harassment that occurs in person, including its detrimental effects on the mental and physical health of women, little is known about the mental effects of TFSH. Furthermore, past TFSH research has often focused on adolescent populations (e.g., Reed et al., 2019), meaning that there is still little understanding as to its impact on young adult women in particular. In examining the broader term of TFSV, studies have found it to be related to symptoms of depression, anxiety, and posttraumatic stress (Cripps & Stermac, 2018; Patel & Roesch, 2020; Snaychuk & O’Neill, 2020). In another study examining “sexting,” which the authors defined as the receipt of sexually explicit images through text messaging, researchers also found that TFSV was associated with increased symptoms of depression, anxiety, and stress when the “sexts” were unwanted or coerced (Klettke et al., 2019). Additionally, research on TFSV in intimate partnerships has found that women experience a variety of consequences, including fear and isolation (Afrouz, 2021). However, research has yet to examine psychological functioning in the context of TFSH and specifically among young adult women.

## Objectification Theory

Although research on objectification theory, as posited by Fredrickson and Roberts (1997), has not yet extended to research on TFSH, work that has been done on in-person contexts can be useful for understanding how it may be related to this new area of research. Following repeated exposure to experiences that treat one as a sexual object rather than a human entity, experiences such as sexual harassment, women victims can start to internalize the view that they are an object that exists solely for the pleasure of others (Fredrickson & Roberts, 1997). This process of self-objectification after sexual objectification can be thought of as a cognitive process wherein the victim locks onto the view of outsiders (Riva et al., 2015). The objectification of one's self then leads to behaviors such as body surveillance which, according to Fredrickson and Roberts (1997), is the “habitual monitoring of the body's outward appearance” (p. 180), because a woman's self-concept is centred on her body. Research onobjectification theory showcases the link between experiences of sexual objectification and self-objectification. In their research on sexual harassment and other forms of sexual violence from strangers that occurred in person, Fairchild and Rudman (2008) found that sexual objectification was associated with self-objectification in their sample of 228 women.

Objectification processes have been theorized and studied in numerous sexualizing contexts in the literature, and though some research has indicated that it may also occur for men (e.g., Wiseman & Moradi, 2010), women have overwhelmingly been the subject of these investigations (Roberts et al., 2018). This is for good reason, as the bodies of women are more often subjected to sexualization, particularly in patriarchal societies where they are seen as commodities or sexual objects. The literature demonstrates that these processes increase vulnerability to psychological symptoms (Roberts et al., 2018). This is of particular concern in technological platforms, where women may not only become more targeted but also more sexualized. Overall, the work of Fredrickson and Roberts (1997) and others on self-objectification in women outlines how it can play a key role in the development of psychological symptoms, such as eating pathology and sexual dysfunction after women experience sexual objectification. However, in the context of psychological functioning and TFSH, self-objectification has not been investigated.

## Psychological Functioning

### Eating behaviors

In their research on female students, Tiggemann and Williams (2012) provided support for the association of self-objectification with disordered eating, as mediated by self-surveillance and other factors. Through structural equation modeling the researchers found an acceptable level of fit of their data to their theorized model, whereby self-objectification was associated with self-surveillance; this was then associated with body shame and appearance anxiety, which were finally associated with disordered eating. For disordered eating, these authors were examining behaviors such as drive for thinness, body dissatisfaction, and bulimic behaviors (Tiggemann & Williams, 2012). Other research by Calogero (2009) and Muehlenkamp and Saris-Baglama (2002) has also demonstrated support for this association. In their work on the objectification theory, Fredrickson and Roberts (1997) outline how eating behaviors can result from a woman's attempt to control their body after it is repeatedly sexualized. Hence, it is important that self-objectification is also examined for a mediating role (i.e., explanatory) in the relationship between TFSH and pathological eating symptoms.

### Substance use

Though Fredrickson and Roberts (1997) did not provide any commentary on objectification processes and substance use in their development of objectification theory, recent research has highlighted the presence of these associations. For instance, research by Carr and Szymanski (2011) indicates that self-objectification may mediate, or help to explain, the association between various forms of in-person sexually objectifying experiences, including sexual violence and substance use. Here, the researchers recruited 289 young women to examine whether sexual objectification was associated with substance abuse both directly and indirectly through self-objectification, body shame, and depression. Their findings indicated support for both direct and indirect associations, with more sexually objectifying experiences being associated with higher self-objectification, which was associated with higher body shame and depression, and finally with higher substance abuse. Carr and Szymanski (2011) discuss their findings by explaining that after sexual objectification, negative mood states resulting from decreased power and control over one's body may lead the women victims to abuse substances. Therefore, given this research by Carr and Szymanski (2011) supporting self-objectification as a potential mediator between in-person sexual harassment and substance use, it may also play a mediating role in the relationship between TFSH and substance use.

### Sexual functioning

Although research by Tiggemann and Williams (2012) showcased that self-objectification was associated with eating symptoms, it also indicated that self-objectification was associated with sexual functioning, as mediated by self-surveillance and appearance anxiety. To measure sexual function, the authors asked their women participants about different aspects of sexual desire, arousal, orgasm, and satisfaction (Tiggemann & Williams, 2012). Fredrickson and Roberts (1997) explain this association within their framework for objectification theory. The authors state that experiencing self-objectification leads to increased mental exertion over monitoring one's body which impedes any investment in rewarding or satisfying ventures, such as sexual activities. This exertion impairs a woman's ability to be mentally present in sexual activity and thereby enhances the likelihood of sexual dysfunction (Fredrickson & Roberts, 1997). Furthermore, these authors outline how an individual experiencing self-objectification will afford external attention to their body rather than attention to body states such as arousal, which then hinders their ability to experience orgasm. Thus, these results also suggest that self-objectification may help to explain a relationship between TFSH and sexual function.

## The Present Study

The purpose of this study was to expand upon the understanding of psychological symptoms that are associated with TFSH by examining eating pathology, substance use, and sexual functioning in female-identifying young adults. The framework provided by objectification theory was also tested to determine whether there was support for self-objectification as a mediator between TFSH and these psychological symptoms (Fredrickson & Roberts, 1997).

## Hypotheses

It was predicted that self-objectification would be supported as a mediator in the associations between TFSH and eating pathology, TFSH and substance use, and TFSH and sexual function based on the tenets of objectification theory (Fredrickson & Roberts, 1997). As incidents of TFSH increased, it was predicted that self-objectification would also increase, as would eating pathology, substance use, and sexual dysfunction.

## Methodology

### Procedure

Approval for this study was granted by Lakehead University's Research Ethics Board. Participants were recruited from Lakehead University's course credit system and online advertisements between October 2020 to March 2021. As an incentive to participate, participants received course credit or had their names entered into a draw for a $25 CAD gift card to a business of their choice.

### Participants

For this study, the data from 629 female-identifying participants from Canada were used. This sample size is above the estimated 558 participants needed, as taken from the simulations performed by Fritz and MacKinnon (2007) that estimated sample sizes for various tests of mediation, including the percentile bootstrap approach that was used in this study. This estimate reflects a power level of .8 and a small effect size for each path in mediation analysis.

#### Exclusion criteria

Infrequent responding, as measured by the Personality Research Form Infrequency Scale, was used as an exclusion criterion in this study (Jackson, 1984). Participants (*n* = 147) who obtained a score of four or higher or who did not complete the entire scale were excluded from the study because it indicated possible random or careless responding. One participant was also excluded from the study because they did not complete over 80% of the primary scales. Therefore, the final number of participants in the sample reached 481. See Table 1 for participant demographics.

**Table 1.** Participant Demographics.

Characteristic		Sample (*N* = 481)
Mean age (SD)		24.9 (8.4)
Race/ethnicity	African	17 (4%)
	Caribbean	7 (2%)
	East Asian	17 (4%)
	Hispanic/Latinx/Latina/Latino	2 (<1%)
	Indigenous	25 (5%)
	Middle Eastern	3 (<1%)
	Pacific Islander	2 (<1%)
	South Asian	20 (4%)
	White	365 (76%)
	Mixed	15 (3%)
Sexual orientation	Exclusively heterosexual	330 (69%)
	Predominately heterosexual, only incidentally homosexual	48 (10%)
	Predominately heterosexual, but more than incidentally homosexual	33 (7%)
	Equally heterosexual and homosexual	27 (6%)
	Predominately homosexual, but more than incidentally heterosexual	6 (1%)
	Predominately homosexual, only incidentally heterosexual	5 (1%)
	Exclusively homosexual	7 (2%)
	No socio-sexual contacts or reactions	6 (1%)
Highest education	Elementary school	1 (<1%)
	Some high school	5 (1%)
	High school completed	99 (21%)
	Some college or technical school	25 (5%)
	College or technical school completed	45 (9%)
	Some undergraduate training	196 (41%)
	Undergraduate degree completed	72 (15%)
	Some graduate or professional training	13 (3%)
	Master's degree completed	19 (4%)
	Doctoral or professional degree completed	6 (1%)
Employment status	Employed full-time	115 (24%)
	Employed part-time	226 (47%)
	Unemployed	140 (29%)
Marital status	Single	205 (43%)
	Married/common-law	97 (20%)
	Separated/divorced	15 (3%)
	Widowed	2 (<1%)
	In a committed relationship	159 (33%)
Student status	Full-time	319 (66%)
	Part-time	26 (5%)
	Not a student	136 (28%)

### Data Collection

SurveyMonkey was used to collect the data from participants, and participants were able to complete the survey at a time and place of their choosing.

#### Technology-facilitated sexual harassment

The TFSH Scale measures the receipt of unwanted sexual attention through technological means and was developed in this study through a deductive method (Boateng et al., 2018). Three primary dimensions of the TFSH domain were identified through a literature review, which included the sending of unwanted sexual material, unwanted sexual comments, remarks, or questions, and unwanted sexual requests. Furthermore, dimensions related to who was identified in the content of the unwanted sexual attention were identified, which included the sender/perpetrator of the TFSH, the victim of the TFSH, or a third party. Powell and Henry's (2019) TFSV Victimization Scale was also used to inform the items. Their seven scale items specific to TFSH were modified for use, such as the item that asks respondents whether they have received “unwanted sexually explicit images, comments, emails, or text messages.” To better capture the dimensions of TFSH behaviors, this item was separated into multiple items. Additional items were added to the scale from other past research on TFSH, such as from Schenk's (2008) work on the Cyber-Sexual Experiences Questionnaire, to target a broad range of TFSH behaviors.

In total, 6 items were included that targeted unwanted sexual material, 5 items were included that targeted unwanted sexual requests, and 16 items were included that targeted unwanted sexual comments or questions. An additional two items were added to include content on unwanted sexual jokes and rumors. All items where an individual who was included in the content of the unwanted sexual attention (i.e., sender, victim, or third party) could be identified were further separated into additional items to capture these different dimensions, based on the recommendation from Reed et al. (2019) to better understand whether TFSH content is personal.

Existing scales for TFSH and TFSV currently use dichotomous scaling to capture whether an incident has occurred. To better capture the frequency of TFSH, participants were asked how frequently they experienced each incident of TFSH on a 5-point Likert-style scale. In terms of TFSH experience over a lifetime, these responses ranged from *never*, *rarely*, *sometimes*, and *often* to *almost always*. Distress ratings were also captured for selected incidents of TFSH; however, due to a similar pattern of results (albeit with smaller effect sizes), these results are not reported here.

#### Psychological Functioning

##### Eating Behaviors

The Eating Attitudes Test (EAT) was used to collect the data on eating behaviors in this study (Garner & Garfinkel, 1979). Although the original version is 40 items in length, the 26-item version of the EAT was used because it has shown comparable psychometric properties to the 40-item version (Garner et al., 1982). The EAT-26 has been shown to measure dieting and avoidance, oral control, and bulimic behaviors (Garner et al., 1982). To increase variability and low-end sensitivity in responses, the EAT was converted from 0-0-0-1-2-3 scaling to 0-1-2-3-4-5 scaling.

##### Substance use

The Alcohol Use Disorders Identification Test (AUDIT) was used to collect information on alcohol use (Babor et al., 2001). This is a 10-item questionnaire that assesses the frequency, dose, and severity of alcohol use (Babor et al., 2001). Scores on the AUDIT range from 0 to 40 and scores above 7 are thought to indicate a higher risk of hazardous alcohol consumption (Babor et al., 2001). In their review of studies examining the psychometric properties of the AUDIT, Reinert and Allen (2007) reported a median internal consistency coefficient of .83, demonstrating the reliability of this test.

##### Sexual function

The Female Sexual Function Index (FSFI) was used to capture information on sexual function (Rosen et al., 2000; Kalmbach et al., 2012). The FSFI measures desire, arousal, orgasm, lubrication, pain, and satisfaction over the past 4 weeks (Rosen et al., 2000). The reliability and validity of the FSFI have been supported, with Cronbach's alphas of .81 and higher, and the scale items also successfully loaded onto their respective factors (Kalmbach et al., 2015). The FSFI was presented with suggested modifications from Boehmer and colleagues (2012), which included the term “vaginal penetration” rather than “sexual intercourse” to account for a wide range of sexual experiences that female participants might have. An additional option of “does not apply to me” was also added to the scale items, where appropriate. In this study, only the desire subscale (i.e., items one and two) was utilized because of uncertainty over participant responses for the other subscales. For instance, most other items on the FSFI included an option for participants to respond that they had not engaged in sexual activity over the past 4 weeks. However, it is unclear whether these responses reflected typical functioning or dysfunction, as participants could be abstaining from sexual activity because they do not have a sexual partner or because they are experiencing issues with arousal, orgasm, lubrication, pain, or satisfaction. Hence, in this study, it was decided that these items would not be included in subsequent analyses.

#### Self-Objectification

Because body monitoring is a manifestation of self-objectification (Fredrickson & Roberts, 1997), the Objectified Body Consciousness Scale (OBCS) (McKinley & Hyde, 1996) was used as a measure of self-objectification. Although this scale contains three subscales (i.e., body surveillance, body shame, and appearance control beliefs), participants in this study were only asked about items from the body surveillance and body shame subscales, which is consistent with past research (e.g., Fairchild & Rudman, 2008; Muehlenkamp & Saris-Baglama, 2002). However, in analyses, only the body surveillance subscale was utilized, which is comprised of items one through eight (see the results section for more information).

#### Response styles

To detect and eliminate potential sources of test-taking error, response bias and nonpurposeful responding were examined in this study. The Desirability Scale and the Infrequency Scale developed and used by Jackson (1984) in his Personality Research Form were used to evaluate these response styles. The Desirability Scale assesses the degree to which participants are responding to the desirability of item content in items such as, “My life is full of interesting activities.” The Infrequency Scale assesses careless or nonpurposeful responding by using items that are infrequently endorsed in the general population, such as “I have never bought anything in a store.”

## Statistical Analyses

The data were examined for accuracy, missing entries, and outliers. Visual inspection of means and standard deviations revealed that all responses were plausible and both pairwise (i.e., correlational analyses), and listwise deletion (i.e., mediation analyses) were utilized to account for missing data. Standardized scores were used to detect univariate outliers using a cutoff of 3.29 (Tabachnick & Fidell, 2013). Nine scores were detected as being larger than 3.29; one of these scores was on the EAT, and the other eight scores were on the AUDIT. Although these potential outliers were not surprising given the large sample size, the outlier on the EAT was altered to a score that was one unit larger than the next most extreme score in the distribution to reduce its effect on analyses (Tabachnick & Fidell, 2013). The outliers for the AUDIT scale were resolved after a transformation of the scale, the details of which can be found in the assumption testing subsection.

### Mediation Analyses

The PROCESS macro (Model 4) for SPSS, as developed by Hayes (2018), was utilized to analyze the mediation models. According to Hayes and Rockwood (2017), the bootstrap confidence interval approach to mediation analysis is a technique that is both rising in popularity among social scientists and is powerful in its ability to detect indirect effects. Unlike other regression-based approaches, such as Baron and Kenny's (1986) causal steps approach, the bootstrap confidence interval relies on quantifying the indirect effect rather than carrying out a set of tests to support its existence (Hayes, 2009). Hence, this study used the percentile bootstrapping approach afforded by the PROCESS macro to test the simple mediation models predicted in this study, with self-objectification as a predicted mediator between TFSH and eating pathology, TFSH and substance use, and TFSH and sexual function.

#### Assumption testing

For normality, residual scatterplots were visually inspected for all variables, and skewness and kurtosis values were examined. Residual scatterplots between each predictor and outcome pair were inspected, and all provided evidence for linearity. However, the residual scatterplots for the TFSH and AUDIT pairing indicated a nonnormal relationship. Unsurprisingly, skewness and kurtosis values for the AUDIT were more than 1, at 1.67 and 2.90, respectively. As a result, the AUDIT scale was transformed using a square root transformation (Tabachnick & Fidell, 2013). A residual scatterplot for this pairing was then re-run, and evidence for normality was present. Furthermore, skewness and kurtosis values were no longer more than ± 1. Residual scatterplots were also visually inspected to reassess whether the assumption of linearity between variables was met. Here, residual scatterplots for predictor-mediator, mediator-outcome, and predictor and mediator-outcome pairings were inspected (Kane & Ashbaugh, 2017). Pairings involving the AUDIT utilized the transformed AUDIT. All residual scatterplots provided evidence for linearity. Additionally, all scatterplots provided evidence for homoscedasticity and normally distributed errors. The Durbin–Watson test was also used to examine whether errors associated with each data point were independent of the errors of other cases (Tabachnick & Fidell, 2013). The test statistics revealed independence of errors, with values ranging from 1.94 to 2.07.

## Results

### Measure Characteristics

The scale means, standard deviations, and internal consistencies are presented in Table 2 for all variables. In Table 2, the psychometric properties of the AUDIT are included based on the subsample of people who endorsed drinking (*n* = 373) because nondrinkers only had to respond to one item. Although the total score for the OBCS scale was initially going to be used for analyses, visual inspection revealed significant overlap between items included in the Body Shame subscale and the EAT. These scales and subscales were empirically evaluated, and not surprisingly, it was determined that the correlations between the Body Shame subscale and the EAT (*r* = .62) and between the total OBCS and the EAT (*r* = .58) were higher than between the Body Surveillance subscale and the EAT (*r* = .40). In past research, the Body Surveillance subscale has been solely utilized to capture self-objectification (e.g., Hanna et al., 2017), and so, because of this lower degree of overlap with the EAT and its use in past research, the Body Surveillance subscale was used in all analyses as a measure of self-objectification.

**Table 2.** Scale Characteristics.

Scale	*M*	*SD*	Internal consistency
Alcohol Use Disorders Identification Test	5.48^a^	4.80^a^	.85^b^
Alcohol Use Disorders Identification Test (transformed)	2.14^c^	0.94^c^	-
Eating Attitudes Test^d^	40.86	20.17	.91
Female Sexual Function Index—Desire Subscale	5.63	2.20	.91
Technology-Facilitated Sexual Harassment (TFSH) Scale	29.09	21.25	.96
Objectified Body Consciousness Scale—Body Surveillance Subscale	37.44	8.37	.84

*Note.* The transformed AUDIT was used instead of the original AUDIT in all analyses.

a Mean and standard deviation is reported for drinkers only. For all participants (*n* = 477), the mean score was 4.29 and standard deviation was 4.81.

b Internal consistency was reported based on standardized items.

c Mean and standard deviation is reported for drinkers only. For all participants (*n* = 477), the mean score was 1.68 and the standard deviation was 1.21.

d Characteristics for the EAT are reported based on the data that was used in subsequent analyses, which contained a modification of a data point due to an outlier being detected.

Item total correlations for all the TFSH scale items were initially computed to evaluate whether items should be included or excluded. Corrected item- total correlations ranged from .54 to .79, which indicated that the items were moderately to strongly correlated with the corrected total score. Results showed that Cronbach's alpha would not increase if any one item was removed, so all items were retained.

### Bivariate Analyses

Bivariate correlations were conducted between TFSH and the measures of psychological functioning to determine the associations of TFSH with eating behaviors, substance use, and sexual functioning. To control for type I error, a Bonferroni correction was applied to each correlation. For bivariate analyses in this study, six separate correlational tests were performed, which yielded an alpha level of .008 with the Bonferroni correction (.05/6). Three correlations were run for frequency ratings of TFSH and three for distress ratings of TFSH. However, as previously mentioned, analyses associated with distress ratings are not reported here due to similar results being obtained as frequency ratings. As is shown in Table 3, all correlations between the measures of psychological functioning and frequency ratings of TFSH were significant at *p* = .008. These correlation coefficients indicate small to moderate relationships between the variables (Cohen, 1988). These correlational analyses were also repeated with social desirability as a covariate to determine if socially desirable answering styles may account for the presence or absence of these associations (Jackson, 1984). All the correlations were observed to remain in the same positive direction while controlling for social desirability. This suggests that the results of the bivariate correlations were not influenced by socially desirable response styles.

**Table 3.** Correlations for Psychological Functioning Variables With TFSH.

		95% confidence interval	
Scale	*R*	Lower	Upper
Eating Attitudes Test	.30*	.20	.39
Alcohol Use Disorders Identification Test	.22*	.12	.31
Female Sexual Function Index—Desire Subscale	.24*^a^	.14	.33

a Higher scores on the Female Sexual Function Index—Desire Subscale indicate increased sexual desire. Here, a positive correlation indicates that as TFSH scores increased, so did sexual desire.

*p < .008.

### Mediation Analyses

A Bonferroni correction yielding an alpha level of .008 was also utilized for the mediation analyses.

#### Eating symptoms

**Figure 1.**
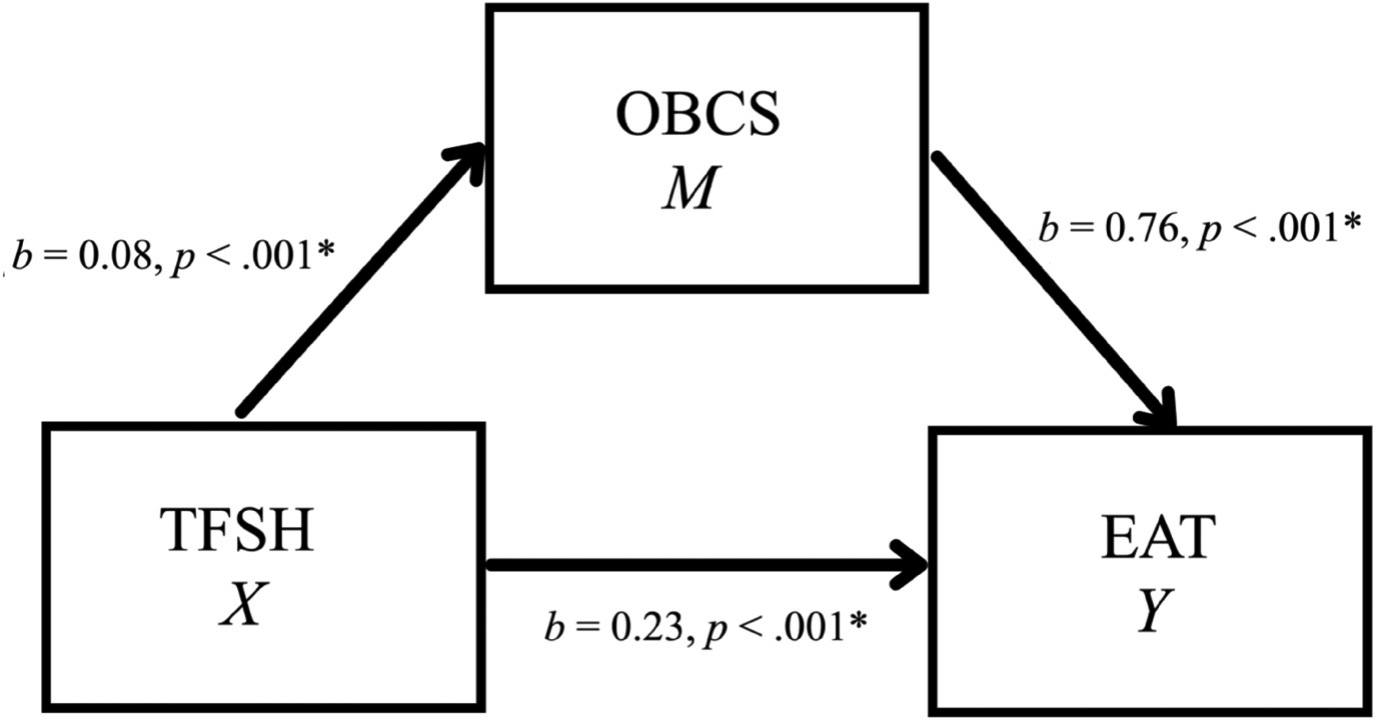
Simple mediation model for the effects of TFSH on EAT as mediated by OBCS.

*Note.* X = predictor variable; M = mediator variable; Y = outcome variable. TFSH = Technology-Facilitated Sexual Harassment Frequency Scale; OBCS = Objectified Body Consciousness Scale—Body Surveillance Subscale; EAT = Eating Attitudes Test. *p < .008 as per the Bonferroni correction.

The first mediation model investigated the hypothesis that self-objectification (OBCS—Body Surveillance Subscale) would mediate the relationship between TFSH and eating symptoms (EAT) (see Figure 1). There was a significant indirect effect of TFSH on eating symptoms through OBCS, *b* = 0.06, 95% confidence interval (CI) [0.03, 0.10] (see Table 4). As a measure of effect size, the partially standardized indirect effect was 0.003, CI [0.001, 0.005]. This result provides evidence of OBCS mediating the relationship between TFSH and eating symptoms.

**Table 4.** Model Coefficients for the Effects of TFSH on EAT as Mediated by OBCS.

		*M* (OBCS)				*Y* (EAT)		
		*b*	*SE*	*p*		*b*	*SE*	*p*
*X* (TFSH)	*A*	0.08	0.02	< .001	*c*’	0.23	0.04	< .001
*M* (OBCS)		_	_	_	*b*	0.76	0.11	< .001
		*R*^2^ = .04 *F*(1, 383) = 17.04, *p* = < .001				*R*^2^ = .19 *F*(2, 382) = 45.11, *p* = < .001		
		

*Note. N* = 385. TFSH = Technology-Facilitated Sexual Harassment Frequency Scale; OBCS = Objectified Body Consciousness Scale—Body Surveillance Subscale; EAT = Eating Attitudes Test.

#### Alcohol use

**Figure 2.**
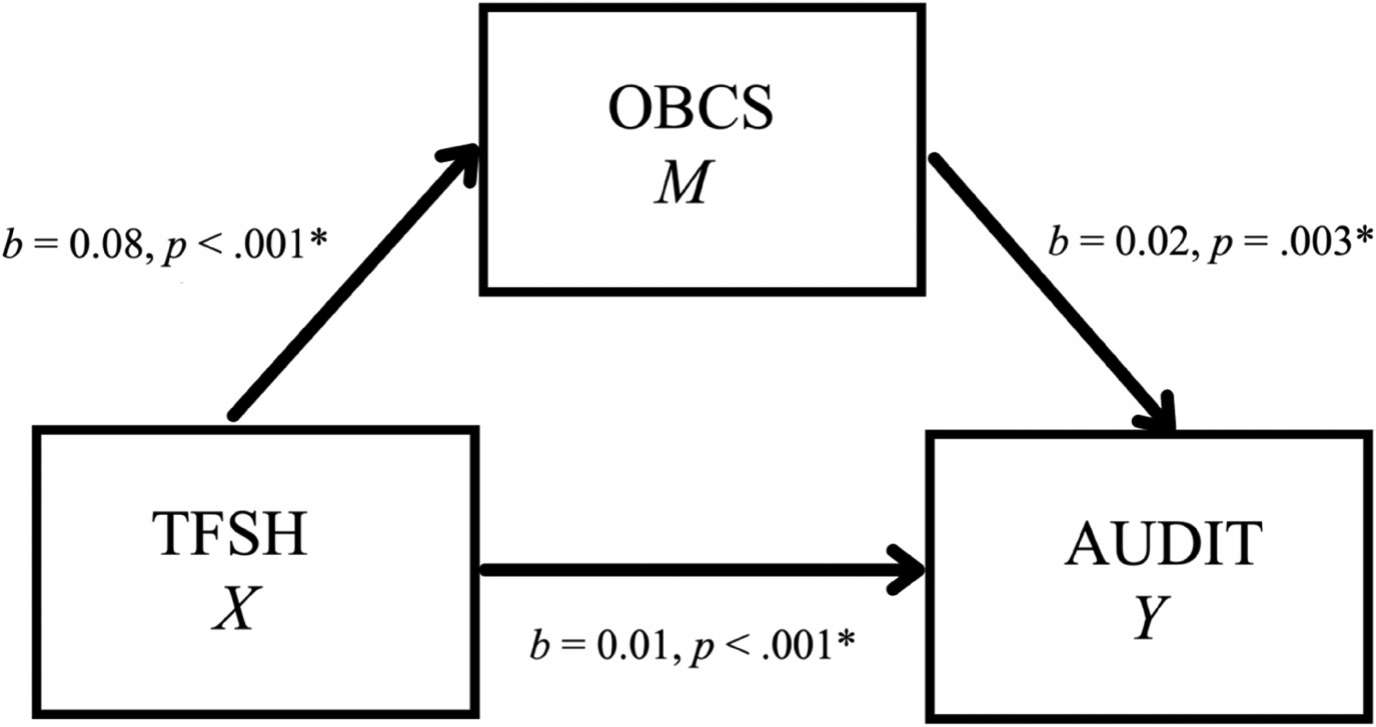
Simple mediation model for the effects of TFSH on AUDIT as mediated by OBCS.

*Note.* X = predictor variable; M = mediator variable; Y = outcome variable. TFSH = Technology-Facilitated Sexual Harassment Frequency Scale; OBCS = Objectified Body Consciousness Scale—Body Surveillance Subscale; AUDIT = Alcohol Use Disorders Identification Test. *p < .008 as per the Bonferroni correction.

The second mediation model examined whether self-objectification (OBCS—Body Surveillance Subscale) would be supported as a mediator between TFSH and alcohol use (AUDIT) (see Figure 2). There was a significant indirect effect of TFSH on alcohol use through OBCS, *b* = 0.002, 95% CI [0.0004, 0.003] (see Table 5). As a measure of effect size, the partially standardized indirect effect was 0.001, CI [0.0003, 0.003]. This result provides evidence for self-objectification functioning as a mediator in this relationship.

**Table 5.** Model Coefficients for the Effects of TFSH on AUDIT as Mediated by OBCS.

		*M* (OBCS)				*Y* (AUDIT)		
		*b*	*SE*	*p*		*b*	*SE*	*p*
*X* (TFSH)	*a*	0.08	0.02	< .001	*c*’	0.01	0.003	< .001
*M* (OBCS)		_	_	_	*B*	0.02	0.01	.003
		*R*^2^ = .04 *F*(1, 397) = 16.14, *p* = < .001				*R*^2^ = .07 *F*(2, 396) = 14.79, *p* = < .001		
		

*Note. N* = 399. TFSH = Technology-Facilitated Sexual Harassment Frequency Scale; OBCS = Objectified Body Consciousness Scale—Body Surveillance Subscale; AUDIT = Alcohol Use Disorders Identification Test.

#### Sexual Function

**Figure 3.**
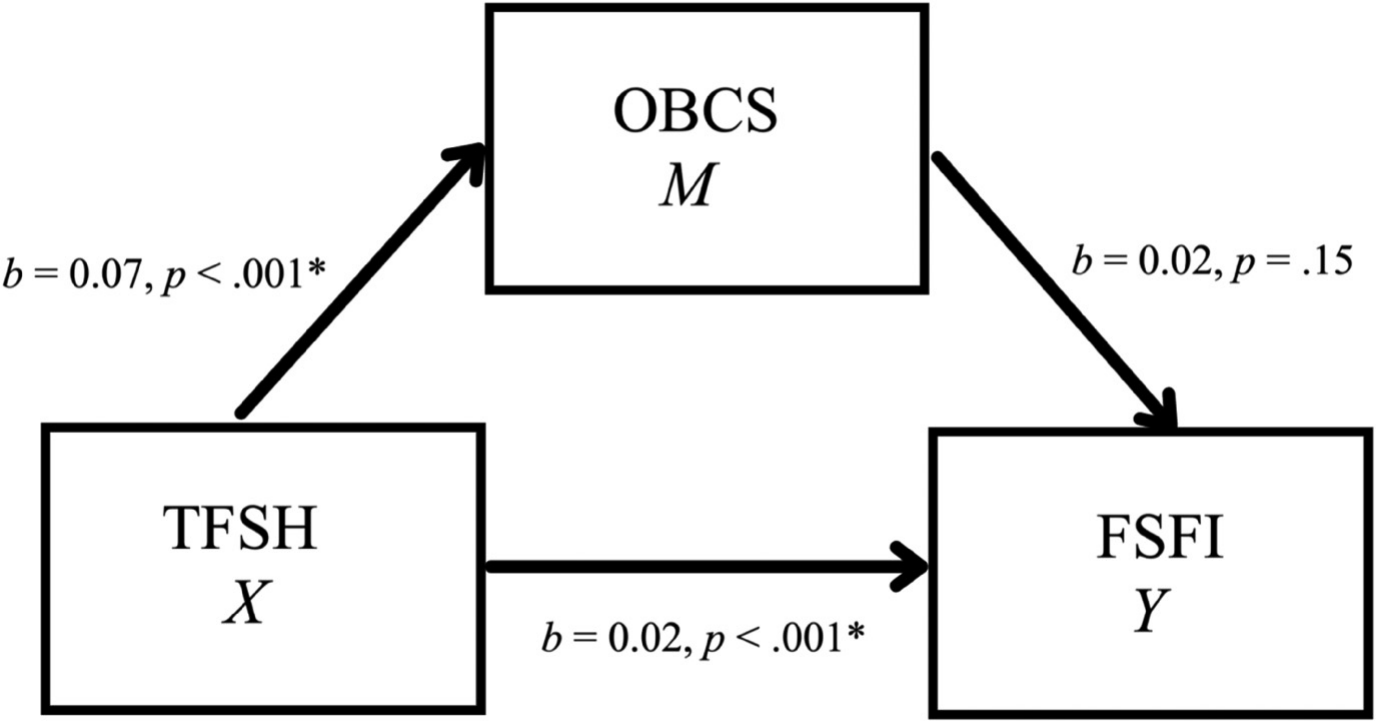
Simple mediation model for the effects of TFSH on FSFI as mediated by OBCS.

*Note.* X = predictor variable; M = mediator variable; Y = outcome variable. TFSH = Technology-Facilitated Sexual Harassment Frequency Scale; OBCS = Objectified Body Consciousness Scale—Body Surveillance Subscale; FSFI = Female Sexual Function Index—Desire Subscale. *p < .008 as per the Bonferroni correction.

The third mediation model investigated whether self-objectification (OBCS—Body Surveillance Subscale) functioned as a mediator in the relationship between TFSH and sexual functioning (i.e., increased sexual desire; FSFI-Desire Subscale) (see Figure 3). There was no significant indirect effect of TFSH on sexual functioning through self-objectification, *b* = 0.001, 95% CI [−0.0006, 0.004] (see Table 6). Hence, self-objectification was not supported as a mediator in this relationship. However, TFSH was a significant predictor of both OBCS (*b* = 0.07, *p* < .001) and FSFI (*b* = 0.02, *p* < .001).

**Table 6.** Model Coefficients for the Effects of TFSH on FSFI as Mediated by OBCS.

		*M* (OBCS)				*Y* (FSFI)		
		*b*	*SE*	*p*		*b*	*SE*	*p*
*X* (TFSH)	*a*	0.07	0.02	< .001	*c*’	0.02	0.01	< .001
*Y* (OBCS)		_	_	_	*b*	0.02	0.01	.15
		*R*^2^ = .03 *F*(1, 393) = 13.91, *p* = < .001				*R*^2^ = .06 *F*(2, 392) = 12.66, *p* = < .001		
		

*Note. N* = 395. TFSH = Technology-Facilitated Sexual Harassment Frequency Scale; OBCS = Objectified Body Consciousness Scale—Body Surveillance Subscale; FSFI = Female Sexual Function Index—Desire Subscale.

## Discussion

TFSH is a problematic consequence of increasing social connection through technology, particularly for young women, though it is a relatively new topic in the literature (Henry & Powell, 2018). In this study, TFSH was explored through a cross-sectional design involving female participants living in Canada. To better understand its relationship to psychological dysfunction, self-objectification was included as a potential mediator. As per objectification theory, the rationale was that experiencing TFSH would increase the likelihood that women would view themselves as an object and consequently experience symptoms of psychological dysfunction (Fredrickson & Roberts, 1997). This theory is particularly relevant to women because of the sexualization of their bodies that is inherent in TFSH and other forms of violence against women. In these mediation models, self-objectification was supported as a mediator between TFSH and both pathological eating symptoms and alcohol use. However, these effect sizes were small, and self-objectification was not supported as a mediator in the relationship between TFSH and sexual functioning. These findings can help shed light on a common phenomenon that little is known about (Henry & Powell, 2018).

### The Role of Self-Objectification

This study is the first to test objectification theory in the context of TFSH and psychological functioning. As was outlined previously, objectification theory outlines how the sexualization of women's bodies can cause them to internalize these messages, which in turn can produce psychological symptoms (Fredrickson & Roberts, 1997). Simple mediation models were used to examine these relationships, and although these models tend to oversimplify complex processes, they can be useful in examining relationships for new phenomena, such as TFSH (Hayes, 2018).

#### Self-Objectification and eating pathology

The relationship between self-objectification and eating pathology has been well-documented in past research (Calogero, 2009; Muehlenkamp & Saris-Baglama, 2002; Tiggemann & Williams, 2012). With respect to the relationship between TFSH and pathological eating symptoms observed in this study, self-objectification, as measured by body surveillance, was supported as a mediator. This indicates that self-objectification may help to explain this relationship. All path coefficients in the models were also in the hypothesized positive direction, meaning that as the frequency of TFSH in women was higher, so were self-objectification and eating pathology. This model provides support for objectification theory, whereby women whose bodies are sexualized may attempt to exert control over their bodies through the development of pathological eating behaviors (Fredrickson & Roberts, 1997).

Though self-objectification was supported as a mediator, these results do not warrant the exclusion of other explanations for the role that self-objectification may play in this relationship. Due to the cross-sectional nature of this study, which prevents any inference about causality, it is completely plausible that self-objectification does not function as a mediator in these relationships but instead functions as a predictor or outcome variable. Other variables may also affect TFSH that might instead be related to self-objectification and eating pathology. For example, it could be that time spent using technology, which could affect the likelihood of TFSH occurring, causes self-objectification and subsequently lead to the development of eating pathology. Furthermore, because the effect sizes of these models were small, the clinical significance of these findings remains uncertain. A large sample size can also lead to the detection of indirect effects in mediation analysis, so this should be a further consideration when interpreting these potentially spurious results (Rucker et al., 2011). A direct effect of TFSH on eating pathology was also supported in the model, suggesting that there is a portion of the relationship between TFSH and eating pathology that is not explained by self-objectification (Zhao et al., 2010). This may signal that another mediating factor is involved, such as lack of awareness of one's body, which has been investigated in other research (Tiggemann & Williams, 2012).

#### Self-Objectification and alcohol use

Self-objectification was also supported as a mediator in the relationship between TFSH and alcohol use. Furthermore, similar to the mediation model involving eating pathology, these path coefficients were all in the hypothesized positive direction. These results provide support for the extension of objectification theory proposed by Carr and Szymanski (2011) that sexualizing events, such as TFSH, can cause women to internalize these experiences and then cope by engaging in alcohol use (Fredrickson & Roberts, 1997). Similar to the mediation results for eating pathology, there are also alternative explanations for the role that self-objectification and other variables may play in these models, whether through a different place in the causal sequence or through confounding effects. For instance, past research has indicated that alcohol and drug use may increase vulnerability to sexual violence (Basile & Smith, 2011). It could be that alcohol use leads to TFSH, which then leads to self-objectification. It should also be noted that the effect sizes were relatively small in this model as well, meaning that the clinical significance of these findings is unclear and likely require more attention in future research. A direct effect of TFSH on alcohol use was also significant in this study, suggesting that some portion of this relationship is not explained by self-objectification (Zhao et al., 2010). This may signal that other potential mediating variables, such as depressive symptoms which has been investigated in work by Carr and Szymanski (2011), may also be involved. Nonetheless, the consistency of these results with the results obtained for TFSH and eating pathology provides more support for the role of objectification processes in TFSH among women.

#### Self-Objectification and sexual function

The final mediation model examined self-objectification as a mediator in the relationships between TFSH and sexual functioning. Here, sexual desire was used as the measure of sexual function. However, self-objectification was not supported as a mediator in this model, which opposes what was hypothesized. Furthermore, self-objectification was not a significant predictor of sexual functioning. This suggests that self-objectification may not explain a potential relationship between TFSH and sexual functioning, which is also in opposition to what has been theorized by Fredrickson and Roberts (1997). The use of sexual desire as the measure of sexual function in this study may have prevented this mediation model from being supported. For instance, other measures of sexual function, such as arousal and orgasm, have been suggested to be involved in objectification processes (Fredrickson & Roberts, 1997). Furthermore, in Tiggemann and Williams’ (2012) study on self-objectification, eating pathology, and sexual functioning, the researchers found that their model had a good fit for eating pathology but a poor fit for sexual functioning. As sexual functioning was measured using desire, arousal, orgasm, and sexual satisfaction, they recommended that researchers examine other aspects of sexual function, such as relationship satisfaction, in future research (Tiggemann & Williams, 2012). However, it is not clear how this might be a better measure of sexual functioning.

In this mediation model, all path coefficients were in a positive direction, meaning that as TFSH frequency ratings increased, so did self-objectification and sexual functioning scores. This is in opposition to what was hypothesized because TFSH and self-objectification were hypothesized to be negative predictors of sexual function. A direct effect was also supported in the relationship between TFSH and sexual function. This might indicate that a mediator other than self-objectification exists that helps to explain this specific relationship (Zhao et al., 2010). For example, in the literature on hypersexuality, emotion dysregulation has been theorized as a causal factor (Garofalo et al., 2016). Perhaps emotion regulation may help to explain the relationship between TFSH and sexual desire or even function as a moderating variable. Other components of the objectification process, such as appearance anxiety, should also be examined in this relationship because they may serve as better measures of self-objectification (Fredrickson & Roberts, 1997; Tiggemann & Williams, 2012). It would be useful if these relationships between TFSH, self-objectification, and sexual functioning were further explored to deduce whether TFSH is related to this aspect of psychological functioning and, with the investigations of other components (e.g., relationship satisfaction) of sexual functioning, whether this relationship might be explained by self-objectification in women.

Overall, this study provides preliminary evidence of relationships between TFSH and eating pathology and alcohol use, as well as between TFSH and sexual function (i.e., increased sexual desire). Furthermore, mediation models investigating the role of objectification processes in these relationships were further supported, specifically in the cases of eating pathology and alcohol use. However, the clinical significance of these findings is uncertain. The role of other variables, as well as the way in which researchers are measuring sexual functioning, are likely important considerations in future research.

### Limitations

Although this study has many strengths, several limitations should be considered when interpreting the findings. The use of a cross-sectional design allowed for the collection of a large sample; however, it prevented any conclusions from being made about the causal relationships between TFSH and psychological functioning. Missing data from the participants in this study were treated with a deletion strategy, and as a result, participants who were not retained in analyses due to missing data may have constituted a specific group of women who are no longer accounted for in the results. For instance, individuals who had missing data may have had less desire to report on their experiences of TFSH because of a higher degree of distress that they experienced. Consequently, this would mean that the study did not capture experiences at the upper end of distress level and, therefore, findings would not be applicable to women who experience a higher degree of distress from incidents of TFSH. Furthermore, the sample consisted of a high proportion of university women, meaning that the results may not be applicable to other groups of women.

Self-report was the method of data collection that was relied upon in this study because of ease and convenience. It also allowed for women to speak on their own experiences of TFSH, which the participants may have found empowering. Although participants may have had trouble recalling incidents of TFSH over their lifetime which is a limitation, to combat some of the limitations that occur from self-report, such as response bias and error, social desirability was controlled for in this study. Participant data that captured information pertaining to other elements of sexual function (e.g., arousal) was also not used in this project. This prevents a more fulsome understanding of sexual functioning in the context of TFSH.

### Future Directions

Cross-sectional designs and convenience samples have been oversaturating the research on TFSV (Henry & Powell, 2018). Therefore, future research might benefit from employing longitudinal designs to examine both TFSH and TFSV. For instance, a study that examined psychological symptoms over time as individuals experience TFSH could provide a better understanding as to whether these experiences do affect one's psychological health. There is also an abundance of quantitative research on TFSH, signalling that more qualitative scholarship on the topic might help to better understand individual experiences. Other aspects of sexual functioning could also be explored in future research. Finally, future research that focused on minority groups would be extremely important to deduce unique experiences of TFSH based on differing intersections of identity (Crenshaw, 1989).

In terms of mental health efforts, Szymanski and colleagues (2011) speak to the need for providers to assess for sexually objectifying experiences, such as TFSH, in their clients. In fact, Szymanski and colleagues (2011) state that research that examines the mediators (such as self-objectification) between sexual objectifying experiences and psychological functioning can influence mental health intervention efforts. For example, this research can help inform work by researchers such as Liss and Erchull (2015), which supports the use of self-compassion with clients experiencing self-objectification. If future research supports self-objectification as a mediator in the relationships between TFSH and eating pathology and alcohol use, then these could be the focus of these types of mental health intervention efforts. Thus, the acquisition of more knowledge on TFSH can assist in improving mental health interventions for victims of TFSH.

## Conclusions

As one of the first studies to examine psychological functioning and TFSH, the results showcased that TFSH was associated with eating pathology, alcohol use, and increased sexual functioning in young women, although the clinical significance and causal nature of these findings are unknown. Furthermore, this study sheds light on the role of self-objectification as a potential mediator in these relationships, particularly between TFSH and eating pathology, and TFSH and alcohol use. Consequently, this study could provide mental health researchers and clinicians with a gateway to providing mental health support for women victims and survivors.

## References

[bibr1-10778012231177998] Afrouz R. (2021). The nature, patterns and consequences of technology-facilitated domestic abuse: A scoping review. Trauma, Violence & Abuse, 24(2), 913–927. 10.1177/15248380211046752 34582729

[bibr2-10778012231177998] Babor T. F. Higgins-Biddle J. C. Saunders J. B. Monteiro M. G. (2001). The alcohol use disorders identification test: Guidelines for use in primary care. World Health Organization. https://apps.who.int/iris/bitstream/handle/10665/67205/WHO_MSD_MSB_01.6a.pdf;jsessionid=4944F0FE44878EF860B0B1D287EB4E36?sequence=1

[bibr3-10778012231177998] Barak A. (2005). Sexual harassment on the internet. Social Science Computer Review, 23(1), 77–92. 10.1177/089443930427154

[bibr4-10778012231177998] Baron R. M. Kenny D. A. (1986). The moderator–mediator variable distinction in social psychological research: Conceptual, strategic, and statistical considerations. Journal of Personality and Social Psychology, 51(6), 1173–1182. 10.1037/0022-3514.51.6.1173 3806354

[bibr5-10778012231177998] Basile K. C. Smith S. G. (2011). Sexual violence victimization of women: Prevalence, characteristics, and the role of public health and prevention. American Journal of Lifestyle Medicine, 5(5), 407–417. 10.1177/1559827611409512

[bibr6-10778012231177998] Boateng G. O. Neilands T. B. Frongillo E. A. Melgar-Quiñonez H. R. Young S. L. (2018). Best practices for developing and validating scales for health, social, and behavioral research: A primer. Frontiers in Public Health, 6, 149. 10.3389/fpubh.2018.00149 29942800 PMC6004510

[bibr7-10778012231177998] Boehmer U. Timm A. Ozonoff A. Potter J. (2012). Applying the Female Sexual Functioning Index to sexual minority women. Journal of Women's Health, 21(4), 401–409. 10.1089/jwh.2011.3072 PMC3321676 22136340

[bibr8-10778012231177998] Calogero R. M. (2009). Objectification processes and disordered eating in British women and men. Journal of Health Psychology, 14(3), 394–402. 10.1177/1359105309102192 19293301

[bibr9-10778012231177998] Carr E. R. Szymanski D. M. (2011). Sexual objectification and substance abuse in young adult women. The Counseling Psychologist, 39(1), 39–66. 10.1177/0011000010378449

[bibr10-10778012231177998] Cohen J. (1988). Statistical power analysis for the behavioral sciences (2nd ed.). Lawrence Erlbaum.

[bibr11-10778012231177998] Crenshaw K. (1989). Demarginalizing the intersection of race and sex: A black feminist critique of antidiscrimination doctrine, feminist theory and antiracist politics. University of Chicago Legal Forum, 139.

[bibr12-10778012231177998] Cripps J. Stermac L. (2018). Cyber-sexual violence and negative emotional states among women in a Canadian university. International Journal of Cyber Criminology, 12(1), 171–186. 10.5281/ZENODO.1467891

[bibr13-10778012231177998] Cuenca-Piqueras C. Fernández-Prados J. S. González-Moreno M. J. (2020). Face-to-face versus online harassment of European women: Importance of date and place of birth. Sexuality & Culture, 24(1), 157–173. 10.1007/s12119-019-09632-4

[bibr14-10778012231177998] Douglass C. H. Wright C. J. Davis A. C. Lim M. S. (2018). Correlates of in-person and technology-facilitated sexual harassment from an online survey among young Australians. Sexual Health, 15(4), 361–365. 10.1071/SH17208 29852924

[bibr15-10778012231177998] Fairchild K. Rudman L. A. (2008). Everyday stranger harassment and women’s objectification. Social Justice Research, 21(3), 338–357. 10.1007/s11211-008-0073-0

[bibr16-10778012231177998] Fredrickson B. L. Roberts T.-A. (1997). Objectification theory: Toward understanding women’s lived experiences and mental health risks. Psychology of Women Quarterly, 21(2), 173–206. 10.1111/j.1471-6402.1997.tb00108.x

[bibr17-10778012231177998] Fritz M. S. MacKinnon D. P. (2007). Required sample size to detect the mediated effect. Psychological Science, 18(3), 233–239. 10.1111/j.1467-9280.2007.01882.x 17444920 PMC2843527

[bibr18-10778012231177998] Garner D. M. Garfinkel P. E. (1979). The eating attitudes test: An index of the symptoms of anorexia nervosa. Psychological Medicine, 9(2), 273–279. 10.1017/S0033291700030762 472072

[bibr19-10778012231177998] Garner D. M. Olmsted M. P. Bohr Y. Garfinkel P. E. (1982). The eating attitudes test: Psychometric features and clinical correlates. Psychological Medicine, 12(4), 871–878. 10.1017/S0033291700049163 6961471

[bibr20-10778012231177998] Garofalo C. Velotti P. Zavattini G. C. (2016). Emotion dysregulation and hypersexuality: Review and clinical implications. Sexual and Relationship Therapy, 31(1), 3–19. 10.1080/14681994.2015.1062855

[bibr21-10778012231177998] Hanna E. Ward L. M. Seabrook R. C. Jerald M. Reed L. Giaccardi S. Lippman J. R. (2017). Contributions of social comparison and self-objectification in mediating associations between Facebook use and emergent adults’ psychological well-being. Cyberpsychology, Behavior, and Social Networking, 20(3), 172–179. 10.1089/cyber.2016.0247 28263683

[bibr22-10778012231177998] Hayes A. F. (2009). Beyond Baron and Kenny: Statistical mediation analysis in the new millennium. Communication Monographs, 76(4), 408–420. 10.1080/03637750903310360

[bibr23-10778012231177998] Hayes A. F. (2018). Introduction to mediation, moderation, and conditional process analysis: A regression-based approach (2nd Ed.). Guilford Press.

[bibr24-10778012231177998] Hayes A. F. Rockwood N. J. (2017). Regression-based statistical mediation and moderation analysis in clinical research: Observations, recommendations, and implementation. Behaviour Research and Therapy, 98, 39–57. 10.1016/j.brat.2016.11.001 27865431

[bibr25-10778012231177998] Henry N. Powell A. (2018). Technology-facilitated sexual violence: A literature review of empirical research. Trauma, Violence & Abuse, 19(2), 195–208. 10.1177/1524838016650189 27311818

[bibr26-10778012231177998] Jackson D. N. (1984). Personality Research Form manual. Research Psychologists Press.

[bibr27-10778012231177998] Kalmbach D. A. Ciesla J. A. Janata J. W. Kingsberg S. A. (2012). Specificity of anhedonic depression and anxious arousal with sexual problems among sexually healthy young adults. Journal of Sexual Medicine, 9(2), 505–513. 10.1111/j.1743-6109.2011.02533.x 22024317

[bibr28-10778012231177998] Kalmbach D. A. Ciesla J. A. Janata J. W. Kingsberg S. A. (2015). The validation of the female sexual function index, male sexual function index, and profile of female sexual function for use in healthy young adults. Archives of Sexual Behavior, 44(6), 1651–1662. 10.1007/s10508-014-0334-y 25091215

[bibr29-10778012231177998] Kane L. Ashbaugh A. R. (2017). Simple and parallel mediation: A tutorial exploring anxiety sensitivity, sensation seeking, and gender. Quantitative Methods for Psychology, 13(3), 148–165. 10.20982/tqmp.13.3.p148

[bibr30-10778012231177998] Klettke B. Hallford D. J. Clancy E. Mellor D. J. Toumbourou J. W. (2019). Sexting and psychological distress: The role of unwanted and coerced sexts. Cyberpsychology, Behavior, and Social Networking, 22(4), 237–242. 10.1089/cyber.2018.0291 30855187

[bibr31-10778012231177998] Liss M. Erchull M. J. (2015). Not hating what you see: Self-compassion may protect against negative mental health variables connected to self-objectification in college women. Body Image, 14, 5–12. 10.1016/j.bodyim.2015.02.006 25828840

[bibr32-10778012231177998] McKinley N. M. Hyde J. S. (1996). The objectified body consciousness scale: Development and validation. Psychology of Women Quarterly, 20(2), 181–215. 10.1111/j.1471-6402.1996.tb00467.x

[bibr33-10778012231177998] Muehlenkamp J. J. Saris-Baglama R. N. (2002). Self-objectification and its psychological outcomes for college women. Psychology of Women Quarterly, 26(4), 371–379. 10.1111/1471-6402.t01-1-00076

[bibr34-10778012231177998] Patel U. Roesch R. (2020). The prevalence of technology-facilitated sexual violence: A meta-analysis and systematic review. Trauma, Violence & Abuse, 23(2), 428–443. 10.1177/1524838020958057 32930064

[bibr35-10778012231177998] Penny L. (2013). Cybersexism: Sex, gender and power on the Internet. Bloomsbury Publishing.

[bibr36-10778012231177998] Powell A. Henry N. (2019). Technology-facilitated sexual violence victimization: Results from an online survey of Australian adults. Journal of Interpersonal Violence, 34(17), 3637–3665. 10.1177/0886260516672055 27697966

[bibr37-10778012231177998] Reed E. Salazar M. Behar A. I. Agah N. Silverman J. G. Minnis A. M. Rusch M. L. A. Raj A. (2019). Cyber sexual harassment: Prevalence and association with substance use, poor mental health, and STI history among sexually active adolescent girls. Journal of Adolescence, 75, 53–62. 10.1016/j.adolescence.2019.07.005 31344557 PMC6716784

[bibr38-10778012231177998] Reinert D. F. Allen J. P. (2007). The alcohol use disorders identification test: An update of research findings. Alcoholism: Clinical and Experimental Research, 31(2), 185–199. 10.1111/j.1530-0277.2006.00295.x 17250609

[bibr39-10778012231177998] Riva G. Gaudio S. Dakanalis A. (2015). The neuropsychology of self-objectification. European Psychologist, 20(1), 34–43. 10.1027/1016-9040/a000190

[bibr40-10778012231177998] Roberts T.-A. Calogero R. M. Gervais S. J. (2018). Objectification theory: Continuing contributions to feminist psychology. In Travis C. B. White J. W. Rutherford A. Williams W. S. Cook S. L. Wyche K. F. (Eds.), APA handbook of the psychology of women: History, theory, and battlegrounds (pp. 249–271). American Psychological Association.

[bibr41-10778012231177998] Rosen R. Brown C. Heiman J. Leiblum S. Meston C. Shabsigh R. Ferguson D. D'Agostino R. (2000). The female sexual function Index (FSFI): A multidimensional self-report instrument for the assessment of female sexual function. Journal of Sex & Marital Therapy, 26(2), 191–208. 10.1080/009262300278597 10782451

[bibr42-10778012231177998] Rotenberg C. (2017). Police-reported sexual assaults in Canada, 2009 to 2014: A statistical profile. Canadian Centre for Justice Statistics.

[bibr43-10778012231177998] Rucker D. D. Preacher K. J. Tormala Z. L. Petty R. E. (2011). Mediation analysis in social psychology: Current practices and new recommendations. Social and Personality Psychology Compass, 5(6), 359–371. 10.1111/j.1751-9004.2011.00355.x

[bibr44-10778012231177998] Schenk S. (2008). Cyber-sexual harassment: The development of the cyber-sexual experiences questionnaire. McNair Scholars Journal, 12(1), 82–91. https://doi.org/https://scholarworks.gvsu.edu/mcnair/vol12/iss1/8

[bibr45-10778012231177998] Snaychuk L. A. O'Neill M. L. (2020). Technology-facilitated sexual violence: Prevalence, risk, and resiliency in undergraduate students. Journal of Aggression, Maltreatment & Trauma, 29(8), 984–999. 10.1080/10926771.2019.1710636

[bibr46-10778012231177998] Suler J. (2004). The online disinhibition effect. Cyberpsychology & Behavior, 7(3), 321–326. 10.1089/1094931041291295 15257832

[bibr47-10778012231177998] Szymanski D. M. Carr E. R. Moffitt L. B. (2011). Sexual objectification of women: Clinical implications and training considerations. The Counseling Psychologist, 39(1), 107–126. 10.1177/0011000010378450

[bibr48-10778012231177998] Tabachnick B. G. Fidell L. S. (2013). Using multivariate statistics (6th ed.). Pearson.

[bibr49-10778012231177998] Tiggemann M. Williams E. (2012). The role of self-objectification in disordered eating, depressed mood, and sexual functioning among women: A comprehensive test of objectification theory. Psychology of Women Quarterly, 36(1), 66–75. 10.1177/0361684311420250

[bibr50-10778012231177998] Wiseman M. C. Moradi B. (2010). Body image and eating disorder symptoms in sexual minority men: A test and extension of objectification theory. Journal of Counseling Psychology, 57(2), 154–166. 10.1037/a0018937 21133567

[bibr51-10778012231177998] Zhao X. Lynch J. G.Jr. Chen Q. , (2010). Reconsidering Baron and Kenny: Myths and truths about mediation analysis. Journal of Consumer Research, 37(2), 197–206. 10.1086/651257

